# Biological mechanisms and clinical significance of endoplasmic reticulum oxidoreductase 1 alpha (ERO1α) in human cancer

**DOI:** 10.1186/s13046-024-02990-4

**Published:** 2024-03-08

**Authors:** Peng Chen, Amit Sharma, Hans Weiher, Ingo G.H. Schmidt-Wolf

**Affiliations:** 1 Department of Integrated Oncology, Center for Integrated Oncology (CIO), University Hospital Bonn, 3127 Bonn, Germany; 2 Department of Neurosurgery, University Hospital Bonn, 53127 Bonn, Germany; 3 Department of Applied Natural Sciences, Bonn-Rhein-Sieg University of Applied Sciences, 53359 Rheinbach, Germany

**Keywords:** Cancer, ER stress, ERO1α, PDI, Immune escape, Prognosis, Inhibitor

## Abstract

A firm link between endoplasmic reticulum (ER) stress and tumors has been wildly reported. Endoplasmic reticulum oxidoreductase 1 alpha (ERO1α), an ER-resident thiol oxidoreductase, is confirmed to be highly upregulated in various cancer types and associated with a significantly worse prognosis. Of importance, under ER stress, the functional interplay of ERO1α/PDI axis plays a pivotal role to orchestrate proper protein folding and other key processes. Multiple lines of evidence propose ERO1α as an attractive potential target for cancer treatment. However, the unavailability of specific inhibitor for ERO1α, its molecular inter-relatedness with closely related paralog ERO1β and the tightly regulated processes with other members of flavoenzyme family of enzymes, raises several concerns about its clinical translation. Herein, we have provided a detailed description of ERO1α in human cancers and its vulnerability towards the aforementioned concerns. Besides, we have discussed a few key considerations that may improve our understanding about ERO1α in tumors.

## Introduction

The endoplasmic reticulum (ER) in eukaryotic cells is the largest organelle of interconnected membranes with diverse functions, including protein synthesis, transport and folding, lipid and steroid synthesis, calcium storage and crosstalk with other organelles [1]. The ER is classified as rough ER and smooth ER, depending on the presence of ribosomes. The rough ER is defined by the presence of membrane-bound ribosomes and mainly performs functions associated with the biosynthesis of membrane and secretory proteins, including their proper folding and modification. The smooth ER, where ribosomes are absent, is primarily involved in lipid and steroid synthesis, carbohydrate metabolism, and calcium ion storage [1, 2]. However, there is little evidence that the rough ER is excluded from the functions of the smooth ER. For instance, the rough ER is also involved in calcium homeostasis in the ER [3, 4]. With the assistance of chaperones, nascent unfolded proteins from ribosomes are subjected to the ER quality control mechanisms [5]. Qualified proteins are subsequently packaged into vesicles and trafficked to the Golgi apparatus for further processing, while misfolded proteins are degraded in the cytosol through ER-associated degradation (ERAD). ERAD is a process driven by proteasomes whereby misfolded proteins are retrogradely transferred from the ER to the cytosolic proteasomes through channel proteins in an energy-consuming manner [5, 6].

**Fig. 1:**
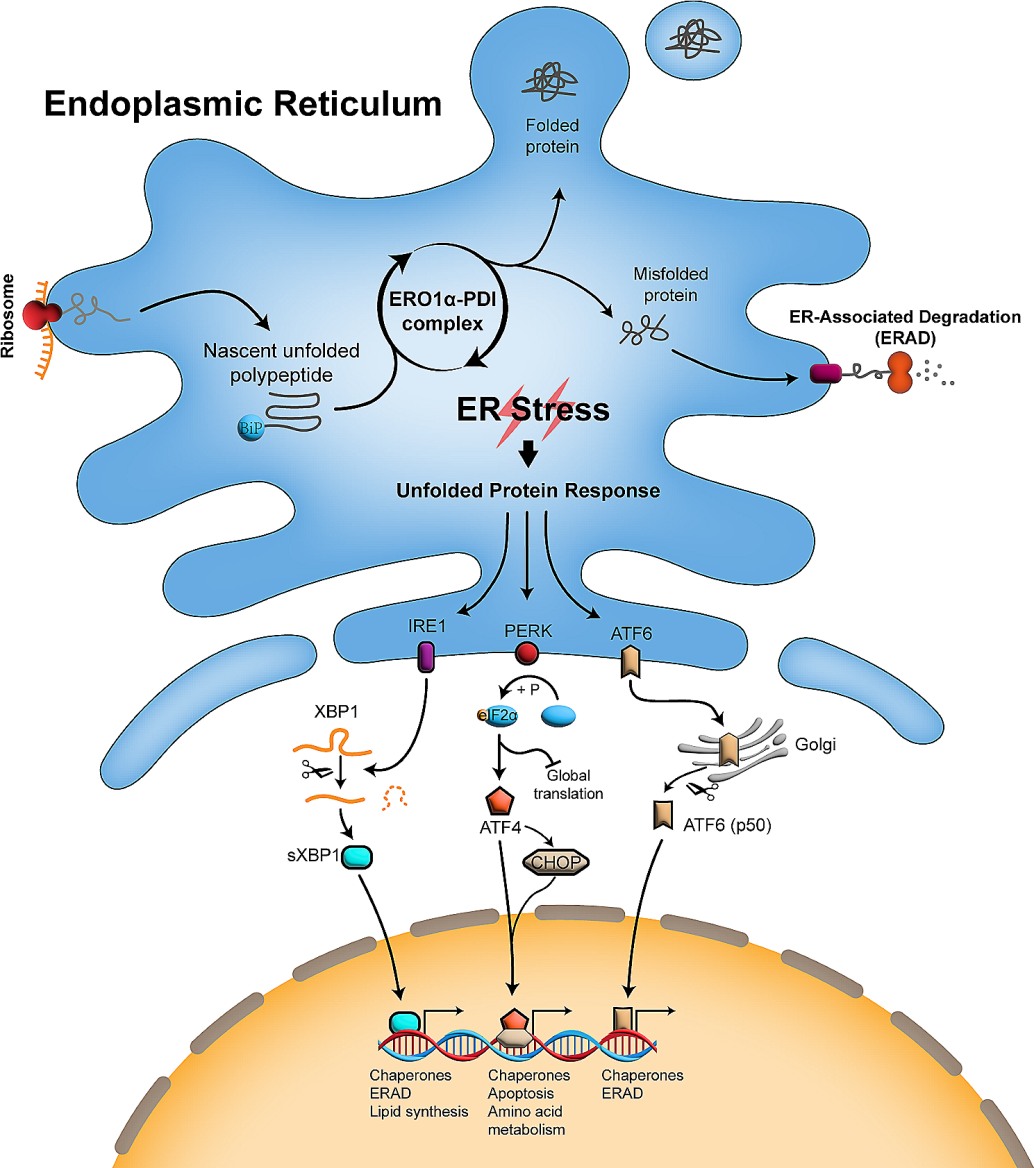
The oxidative protein folding in the ER and the unfolded protein response. Nascent polypeptides from ribosomes are oxidatively folded by the ERO1α-PDI complex. Dysfunctional ERO1α-PDI complex results in accumulation of unfolded and misfolded protein, which then arouses the UPR and sends signals to retard translation and facilitate processing and degradation of protein. During the UPR activation, ATF6 is transported to the Golgi apparatus, where it is processed to its activating form ATF6 (p50) and then locates to the nucleus for ERAD-related gene transcription. Activated PERK phosphorylates eIF2α, leading to global translation inhibition but selectively inducing ATF4. AFT4 then enters into the nuclear and activates gene transcription. Of note, facing the overwhelming ER stress, ATF4 can also activate CHOP, a transcription factor then inducing apoptosis through the caspase pathway. Activation of IRE1 induces the splicing of XBP1 and its activating form sXBP1 then goes into the nucleus and initiates the ERAD-related gene transcription. ERO1α, endoplasmic reticulum oxidoreductase 1 alpha; PDI, protein disulfide isomerase; BiP, binding immunoglobulin protein; ERAD, endoplasmic reticulum (ER)-associated degradation; UPR, unfolded protein response; IRE1, inositol requiring enzyme 1; PERK, protein kinase (PKR)-like ER kinase; ATF6, activating transcription factor 6; eIF2α, eukaryotic initiation factor 2α; XBP1, X-box binding protein 1; sXBP1, spliced XBP1; CHOP, C/EBP homologous protein

Given the complex and pivotal functions, the ER is strictly and intricately regulated to meet cellular biological activities. Protein homeostasis is a distinctive feature of a properly functioning ER, where protein synthesis is compatible with processing [7]. However, when cells are exposed to stressful conditions such as nutrient shortage, hypoxia, calcium dyshomeostasis, and oxidative stress, the protein-folding capacity of cells is disrupted, leading to the accumulation of unfolded and misfolded proteins in the ER lumen, thereby provoking ER stress (Fig. 1) [8]. In reaction to ER dysfunction, cells initiate an adaptive defense mechanism known as the unfolded protein response (UPR) to reinforce protein folding and degradation capacities, ultimately tackling the ER stress and restoring protein homeostasis [9]. Therefore, the UPR is a protective response by which cells to handle ER stress.

The UPR is initiated by three transmembrane sensors: inositol requiring enzyme 1 (IRE1), protein kinase (PKR)-like ER kinase (PERK), and activating transcription factor 6 (ATF6) (Fig. 1) [8, 10]. The accumulation of unfolded and misfolded proteins binds more chaperone proteins, such as glucose-regulated protein 78 (GRP78, also known as binding immunoglobin protein (BiP)), resulting in the dissociation of BiP from these three sensors. Upon BiP release, ATF6 is transported to the Golgi apparatus where it is processed into its active form, ATF6 (p50), and subsequently translocated to the nucleus to promote the transcription of chaperone and ERAD-related genes. Dimerization and autophosphorylation occur when PERK and IRE1 decouple from BiP. Activated PERK in turn phosphorylates eukaryotic initiation factor 2α (eIF2α), which leads to transient inhibition of global protein translation to restore abnormal ER but selectively induces activating transcription factor 4 (ATF4). AFT4 then enters the nucleus and activates the transcription of genes related to chaperone, apoptosis and amino acid metabolism. Activation of IRE1 induces the splicing of X-box protein 1(XBP1) mRNA and then the formation of its active form, sXBP1. sXBP1 is translocated to the nucleus to initiate the transcription of genes responsible for chaperone, ERAD and lipid synthesis. Collectively, UPR contributes to restore ER protein homeostasis by retarding general protein translation and increasing the translation of ER resident chaperones and components of the protein degradative machinery to prevent the aggregation of unfolded and misfolded proteins. Moderate ER stress can be dispelled by proper collaboration among the respective UPR branches, therefore maintaining cell survival, however, persistent or severe ER stress eventually induces cell death [11]. Ample evidence supports that unrelievable ER stress leads to cell apoptosis, and two UPR branches, PERK and IRE1, control cell fate under ER stress [10, 12, 13]. In the face of overwhelming ER stress, ATF4, which is selectively activated in the PERK branch, has been shown to induce apoptosis by both inhibiting the anti-apoptotic protein Bcl-2 [14] and promoting the pro-apoptotic proteins BIM [15] and PUMA [16] through the activation of the transcription factor C/EBP homologous protein (CHOP). On the other hand, IRE1 can offset the apoptosis signals from the PERK/ATF4/CHOP branch by degrading apoptosis-dependent components [10]. However, IRE1 has also been revealed to promote apoptosis and autophagy by activating the c-Jun N-terminal kinase (JNK) pathway [17, 18].

ER stress has been documented in most major types of human cancer, especially in solid tumor [19]. Of importance, amounting evidence has shown that ER stress and the subsequent UPR modulate various pro-tumoral properties, including angiogenesis, metabolism, metastasis, and chemoresistance in cancers, while reprogramming the function of immune cells in the tumor microenvironment (TME) [8, 20, 21]. In addition, ER stress has also been identified in cancer stem cells (CSCs) and dormant tumor cells, which are mostly to blame for relapse, contributing to their stemness maintenance, quiescence and chemoresistance [22–24]. Targeting UPR, the adaptive mechanism of ER stress, induces the differentiation of CSCs, increases cell death and sensitivity to chemotherapy in CSCs and dormant tumor cells [23–25]. Overall, adaptation to ER stress confers a survival advantage to tumor cells, but also renders them vulnerable to environmental perturbations. Therefore, targeting ER stress to disturb the adaptive mechanism has emerged as an attractive approach for cancer immunotherapy in recent years [26, 27].

Oxidative protein folding is one of the critical functions of the ER, and both folding efficiency and quality play crucial roles in inducing UPR. Compared to the cytosol, the redox environment in the ER is oxidative, which favors the formation of disulfide bonds. The oxidative environment in the ER is mainly due to the distribution of reduced/oxidized glutathione (GSH), where the glutathione redox potential (E_GSH_) in the ER is much higher than that of in the cytosol [28, 29]. Endoplasmic reticulum oxidoreductase 1 alpha (ERO1α) has been reported to help maintain the oxidative environment in the ER, as knockout of ERO1α significantly reduced E_GSH_ in the ER [30, 31].

ERO1α (also known as ERO1A or ERO1L) is a flavin adenosine dinucleotide (FAD)-containing ER-resident thiol oxidoreductase responsible for catalyzing disulfide bond formation in nascent polypeptides, working in conjunction with protein disulfide isomerase (PDI) [32]. In recent years, ERO1α has been implicated in various facets of tumor progression, such as tumor growth, angiogenesis, metastasis and chemoresistance, due to its high expression in tumors [33]. Given its function, ERO1α has been reported to promote the oxidative folding of certain tumor-favoring proteins, such as vascular endothelial growth factor (VEGF), programmed cell death ligand-1 (PD-L1), and matrix degrading enzymes (MMPs). In this review, we will delineate the profile of ERO1α in the context of tumors, focusing on four key areas: (1) the structure and function of ERO1α, (2) the expression and regulation of ERO1α, (3) the impact of ERO1α, and (4) targeting ERO1α in tumors.

### Distribution, structure and function of ERO1α

ERO1α in mammals was first reported and characterized in 2000, sharing extensive homology with the *Saccharomyes cerevisiae ERO1* gene and involved in oxidative protein folding in the ER [34]. In mammals, there are two ERO1 isoforms, ERO1α and ERO1β [34, 35]. ERO1α is expressed ubiquitously in all cell types as its crucial role in oxidative protein folding, whereas ERO1β is selectively expressed in pancreatic and stomach cells, indicating its significance in insulin and glucose metabolism [36]. Of note, in addition to oxidative protein folding, ERO1α is also implicated in various biochemical pathways, such as calcium release and regulation of nicotinamide adenine dinucleotide phosphate oxidase (NOX) activity. ERO1α has been reported to trigger calcium release from the ER to the cytosol or mitochondria *via* regulating the inositol 1,4,5-triphosphate receptor (IP3R)- and ryanodine receptor (RyR)-induced calcium release [37–39]. Furthermore, the released calcium activates the enzyme calcium/calmodulin-dependent protein kinase II (CaMKII), which in turn induces NOX expression [40]. In addition, an interesting finding is that ERO1α knockout in mammals is not as fatal as in yeast, and mice with ERO1α knockout exhibit a mere retardation in disulfide bond formation [41]. Indeed, it has been demonstrated that ERO1α function in oxidative protein folding can be compensated by other redundant oxidoreductases, such as peroxiredoxin 4 (PRDX4), glutathione peroxidase 8 (GPx8), peroxiredoxin IV (PrxIV) and vitamin K epoxide reductase (VKOR) [42–45].

**Fig. 2:**
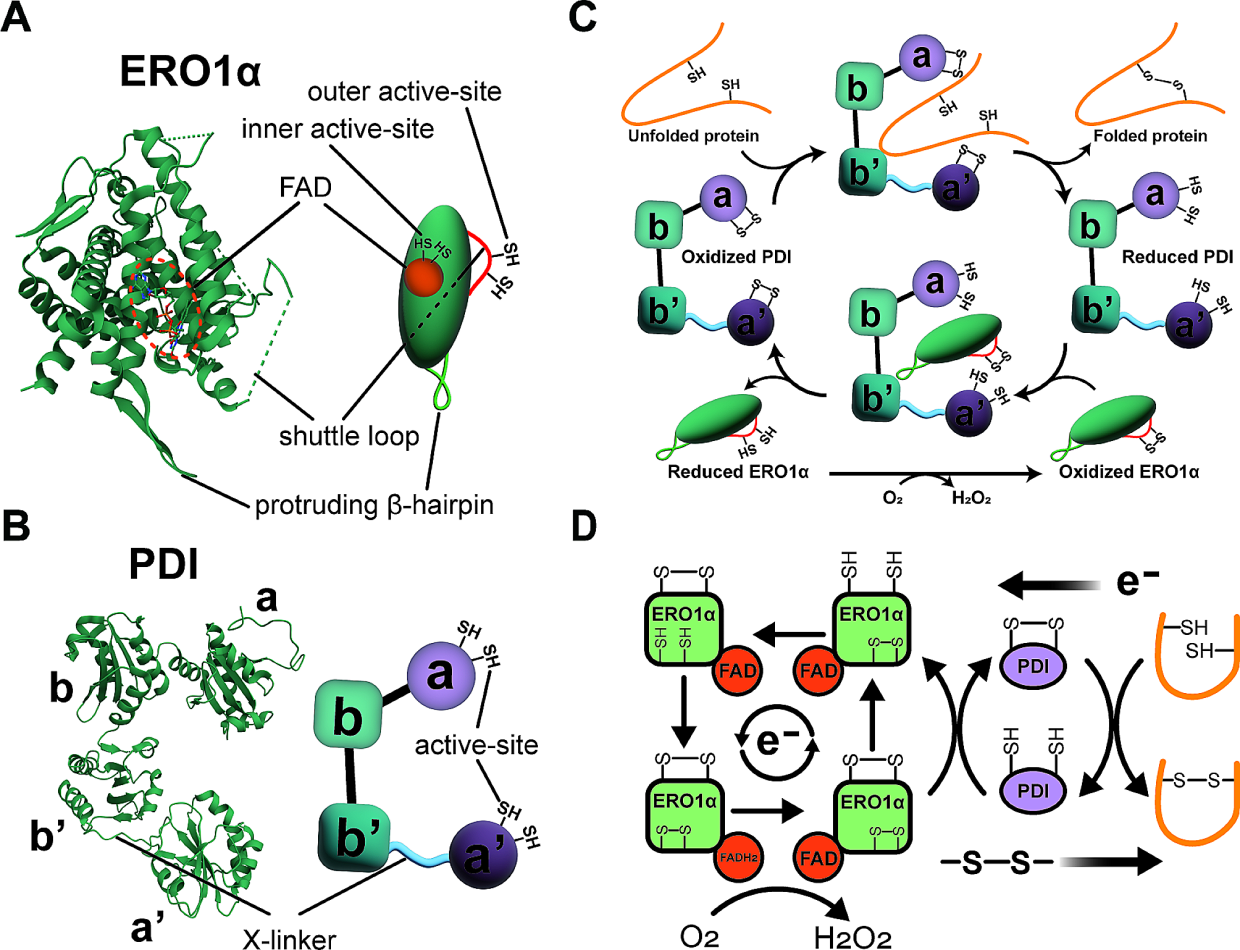
The crystal structure of human ERO1α and PDI and their working flow. The crystal structure and illustrative diagram of ERO1α (PDB: 3AQH) **(A)** and PDI (PDB: 4EKZ) **(B)**. **(C)** The catalytic cycle of the ERO1α-PDI complex. The PDI a’ domain regulates the affinity of PDI to ERO1α and polypeptides by inducing the spatial rearrangement of the a’ and b’ domains through the conformational change of the x-linker region. Oxidized PDI has higher affinity to polypeptides and binds to them via the b’ domain. Oxidizing equivalents are transferred from the active site disulfide bonds of oxidized PDI to the unfolded polypeptides and PDI is therefore reduced. Reduced PDI shows higher affinity to ERO1α. Consequently, polypeptides dissociate from reduced PDI and are displaced by ERO1α. PDI is re-oxidized by ERO1α and then re-enters into a new catalytic cycle. **(D)** The electron transport chain within the oxidative folding. PDI oxidizes cysteines in nascent polypeptides to form disulfide bonds and accepts electrons from polypeptides, resulting in the reduction of PDI. Electrons from PDI are passed onto ERO1α leading to the reduction of the outer active site of ERO1α and the oxidation of PDI. Oxidized PDI then goes into a new round, while the outer active site of ERO1α shuffles electrons to the inner active site and onto the adjacent FAD coenzyme. FAD is reduced to FADH_2_ upon accepting electrons. As the ultimate acceptor, molecular oxygen accepts electron from FADH_2_ with the production of H_2_O_2_. ERO1α, endoplasmic reticulum oxidoreductase 1 alpha; PDI, protein disulfide isomerase; FAD, flavin adenosine dinucleotide

Human ERO1α protein is functionally composed of two regions: a four antiparallel alpha-helices core region containing a binding site for the FAD coenzyme and an adjacent inner active-site, as well as a shuttle loop with an outer active-site (Fig. 2A) [46]. In addition, ERO1α features a protruding β-hairpin responsible for docking with PDI. Human PDI protein consists of two thioredoxin-like redox-active domains (a and a’) and two thioredoxin-like redox-inactive domains (b and b’), and a flexible x-linker between the a’ and b’ domains, in which the b’ domain is the common binding site for polypeptides and ERO1α (Fig. 2B) [47, 48]. The redox state of the PDI a’ domain regulates the affinity of the b’ domain to ERO1α and polypeptides by inducing the spatial rearrangement of the a’ and b’ domains through the conformational change of the x-linker region [49, 50].

ERO1α functions as an exchange center for disulfide bonds and electrons to assist PDI in the *de novo* disulfide bond formation in nascent polypeptides (Fig. 2C and D). Briefly, disulfide bond formation occurs between the sulfhydryl (-SH) side chains of two cysteine residues of substrate proteins. One sulfhydryl in the substrate attacks a disulfide bond in the active site of PDI, creating a transient mixed-disulfide bond. Another sulfhydryl then initiates a nucleophilic attack on this mixed-disulfide bond, resulting in the formation of an intramolecular disulfide bond within the substrate, leaving PDI in reduced state. Reduced PDI requires re-oxidation to allow another round of disulfide bond formation. ERO1α continuously re-oxidizes PDI and transfers disulfide bonds to PDI by reducing molecular oxygen, making the process sustainable. During the process, electrons flow in the reverse direction with disulfide bonds, and ultimately are accepted by molecular oxygen with the production of H_2_O_2_ (Fig. 2D). It has been reported that ERO1α is one of the primarily sources of H_2_O_2_, accounting for 25% of the total H_2_O_2_ in cells [51, 52]. H_2_O_2_ is involved in cell signaling cascades as a secondary messenger and can also be reduced to O_2_ and H_2_O through antioxidant enzyme system, such as superoxide dismutase, catalase, and glutathione peroxidase [53, 54].

Notably, in terms of tumors, especially for solid tumors, hypoxia is a prominent characteristic [55]. Hypoxia retards the formation of disulfide bonds, as molecular oxygen acts as a provider of oxidizing equivalents and an acceptor of electrons [56, 57]. However, some hypoxia-induced pro-tumoral proteins, such as VEGF and carbonic anhydrase 9 (CA9), complete disulfide bond formation and traverse through the secretory pathway in anoxic conditions, suggesting that ERO1α can utilize alternative electron acceptors instead of oxygen [56]. In addition, despite the existence of back-up systems of ERO1α, the disulfide bond formation of some pro-tumoral proteins, such as VEGF, PD-L1, is indeed restrained upon ERO1α inhibition, implying the dysfunction of these compensatory mechanisms in tumor cells [58, 59]. Therefore, tumor cells are more dependent on ERO1α than normal cells, thereby providing an excellent opportunity for the use of ERO1α inhibitors. ERO1α inhibition impairs oxidative protein folding in tumor cells, whereas it has a limited effect on normal cells due to the presence of back-up systems [41, 45, 60, 61]. Furthermore, it remains unclear whether the functions of ERO1α beyond disulfide bond formation are substitutable.

### ERO1α in tumor landscapes

#### Expression profile of ERO1α in diverse tumors

All data from different studies were integrated to Table 1. The expression data consist of tumor cell lines (vs. normal cell lines), human tumor tissues (vs. normal or tumor adjacent tissues) and online gene expression databases (Oncomine, GEO, TCGA and GTEx). According to the consolidated result, except prostate cancer, in which ERO1α expression had no significant difference in both tumor tissues and tumor cell lines compared to their normal counterparts, ERO1α expression was up-regulated in bile duct cancer, cervical cancer, lung cancer, pancreatic cancer, breast cancer, liver cancer and gastric cancer. A pan-cancer expression analysis from the Oncomine database revealed up-regulated ERO1α in 10 cancer types while reduced in esophageal cancer, head and neck cancer, and leukemia [64]. Taken together, these data support that ERO1α is highly expressed in the vast majority of tumors, implying a potential role in tumor biology.

#### Regulation of ERO1α in tumors

In all cases, the interaction with PDI is the fundamental regulation of ERO1α. ERO1α is tightly controlled by intramolecular disulfide bonds and PDI to maintain an equilibrium between reduced and oxidized PDI, ensuring sustainable oxidative protein folding [73–76]. In the UPR process, ERO1α is regulated by CHOP, an activated downstream transcription factor in the PERK branch [77, 78]. Furthermore, the phosphorylation of Ser145 has been reported to greatly enhance ERO1α oxidase activity [31].

In tumor settings, hypoxia is the leading factor for the regulation of ERO1α. Hypoxia is a distinctive feature of the TME, especially in the case of solid tumors [55]. In adapting to hypoxia, tumor cells evolve into an aggressive phenotype, acquiring invasive and metastatic properties, and crafting an immunosuppressive environment [55]. Importantly, hypoxia also induces ER stress and consequently, the UPR [79, 80]. Hypoxia-induced up-regulation of ERO1α has been demonstrated to depend on hypoxia-inducible factor-1 alpha (HIF-1α) [81], which controls the up-regulated transcription of most downstream genes in adaptive responses to hypoxia, and the ablation of HIF-1α resulted in a complete failure to up-regulate ERO1α under hypoxic condition [82]. In an esophageal cancer study, ERO1α was found to be capable of sensing and being post-translationally regulated by the sulfur amino acid precursor homocysteine [83]. The researchers observed that homocysteine induced the active form of ERO1α, suggesting ERO1α might be regulated by antioxidants or redox-active metabolites in epithelial cells. The affinity of ERO1α to amino acid precursor identifies a potential link between diet, antioxidants, and oxidative protein folding in the ER. The transcription factor nuclear factor IB (NFIB) has previously been shown to facilitate tumorigenesis in several cancer types [84–86]. Federica et al. found that NFIB enhanced angiogenesis in breast cancer *via* the ERO1α/HIF-1α/VEGF pathway, in which ERO1α was identified as a direct transcriptional target of NFIB through chromatin immunoprecipitation (ChIP) assay [87]. MicroRNAs, a category of small non-coding RNAs that target mRNA and inhibit their expression, have also been shown to down-regulate ERO1α. Li et al. demonstrated that microRNA-144-3p inhibited tumorigenesis in oral squamous cell carcinoma by down-regulating the ERO1α/STAT3 pathway [88]. In addition, down-regulated microRNA-582-5p and microRNA-218-5p in cervical cancer and lung cancer, respectively, have been shown to promote tumor progression *via* targeting ERO1α [89, 90]. Accumulating evidence has shown that epigenetic modifications play a crucial role in the regulation of gene expression [91]. Using bioinformatic analysis, Liu et al. and Shi et al. found that the promoter methylation of ERO1α was markedly reduced in lung cancer, suggesting that hypomethylation of the promoter relieved transcription inhibition, resulting in the overexpression of ERO1α [64, 67]. In a study focusing on liver cell apoptosis, DNA methyltransferase 1 (DNMT1) and G9a (also known as euchromatic histone-lysine N-methyltransferase 2 (EHMT2)) were shown to be responsible for the reduction of ERO1α by mediating the hypermethylation and H3K9me2 modification of the ERO1α promoter, respectively [92].

Secreted proteins from non-tumor cells can also influence ERO1α in tumor cells. Seungeun et al. reported that tumor associated microphage (TAM) derived C-C chemokine ligand (CCL) 2 induced ERO1α mRNA expression in breast epithelial cells, leading to the upregulation of MMP-9 and an invasive phenotype [93].

Overall, various intrinsic and extrinsic cellular regulators show ability to modulate ERO1α. Indeed, as a vital adaptive mechanism for tumor cells responding to environmental perturbations, the up-regulation of ERO1α contributes to improve the plasticity and survival of tumors. It is worth noting that the current regulatory factors may primarily originate from tumor cells themselves. Nevertheless, the regulatory roles of the interplays between tumor and non-tumor cells, in particular immune cells that modulate these interactions, warrant more attention. Likewise, epigenetic regulation of ERO1α modulation also needs consideration.

### ERO1α in mediating cancer progression and immune escape

#### Biological behavior

Tumor cells evolved from normal ones through precancerous status under the influence of carcinogenic factors [102]. During this process, cells undergo a series of biological events, including initiation, promotion and progression, ultimately acquiring an aggressive phenotype. The malignant potential of tumor cells is typically in vitro assessed by proliferation, migration and invasion assays. The effects of ERO1α on tumor biological behavior across various tumor types have been described in Table 2. In nearly all relevant studies, knockdown (KD) or knockout (KO) of ERO1α impaired the proliferation, migration and invasion of tumor cells, while overexpression (OE) resulted in opposite outcomes. However, one study on breast cancer has shown that knockdown of ERO1α had no significant impact on malignant potential compared to the normal cells. Additionally, two studies demonstrated that ERO1α also inhibited the proliferation of tumor cells by arresting the cell cycle [66, 67].

#### Epithelial-mesenchymal transition (EMT)

EMT is an important feature of tumor cells in pre-metastatic niche [103]. During the EMT, epithelial tumor cells can transform into cells with a mesenchymal phenotype, gradually losing the connection to basement membrane, degrading extracellular matrix (ECM), and increasing invasion abilities. EMT is commonly characterized by the decrease of epithelial cell hallmarks and the increase of mesenchymal cell hallmarks. ERO1α has been shown to promote EMT in lung cancer, liver cancer, colorectal cancer, bile duct cancer and cervical cancer (Table 2). Aside from EMT hallmarks, integrins and MMPs are also crucial molecules in tumor migration, in which integrins are responsible for the adhesion of tumor cells to ECM, while MMPs are major contributors to the degradation of ECM [103, 104]. Hence, the collaboration between integrins and MMPs contributes to the metastatic capacity of tumors. ERO1α has been reported to promote the expression of integrin β1 and MMP2/9 by enhancing the oxidative folding of these proteins [69, 71, 93, 101].

#### Angiogenesis

Angiogenesis is a pivotal factor for tumor growth, metastasis and colonization, as it supplies nutrients and channels for tumor spread [105]. The ERO1α effects on angiogenesis were in vitro and in vivo investigated by human umbilical vein endothelial cells (HUVEC) migration and tube-formation assay and CD31^+^/CD34^+^ staining in human or mouse tumor tissues (Table 2). Studies in breast and liver cancer revealed that ERO1α contributed to promoting the migration and tube formation of HUVEC cells [58, 87, 96], as well as increasing blood vessel density in mouse tumor tissues [58, 87, 94]. Moreover, ERO1α levels were also positively correlated with blood vessel density in human tumor tissues [94]. For the mechanism, current studies indicate that VEGF, a potent angiogenic agent, is the common effector by which ERO1α exerts its pro-angiogenesis role. On the one hand, as a protein with disulfide bonds, VEGF is up-regulated by ERO1α through enhancing its oxidative folding [87, 96]. On the other hand, ERO1α indirectly up-regulates VEGF *via* HIF-1α [87, 94], which is a well-established mediator in VEGF regulation [106]. H_2_O_2_ generated by ERO1α during oxidative folding in the ER freely diffuses into the cytoplasm, where it then stabilizes HIF-1α by inhibiting prolyl hydroxylases (PHDs) [107, 108]. In addition, ERO1α has been reported to modulate VEGF *via* the S1PR1-STAT3 signaling pathway in liver cancer cells [58], and the deficiency of ERO1α in cervical cancer cells impaired the secretion of VEGF due to N-hyper-glycosylation [109].

#### In vivo tumorigenesis

Xenograft models of knockdown/knockout or overexpressing ERO1α tumor cells into mice were employed to investigate the in vivo tumorigenesis of ERO1α. Studies showed that silencing ERO1α resulted in retarded tumor growth, metastasis, and ameliorated overall survival (OS), while the overexpression of ERO1α produced opposite results (Table 2). In a study focusing on breast cancer, knockout of ERO1α did not significantly impact tumor growth compared to wild-type (WT) cells, however, it impeded lung metastasis [96].

#### Immunosuppressive tumor microenvironment (iTME)

TME is a complex ecosystem that contains immune cells, stromal cells, vasculature and ECM. However, immune cells in TME, such as TAM and myeloid-derived suppressor cells (MDSCs), often exhibit an immune-suppressive phenotype due to their “education” through the crosstalk with tumor cells [110]. Current findings indicate that ERO1α has broad and profound influences on TME, contributing to shape an immunosuppressive microenvironment. Analyses demonstrated that ERO1α mRNA levels were negatively correlated with the number of cells that define anti-tumor immunity, such as CD8^+^ T cells, B cells and natural killer (NK) cells, whereas positively correlated with immunosuppressive cells, including cancer-associated fibroblasts (CAFs), MDSCs and TAMs [64, 111].

In terms of tumor cells, which live in a hypoxic microenvironment, the high expression of ERO1α is essential for tumor survival and progression. On the one hand, the high proliferation rate and crosstalk with non-tumor cells require a strong demand of protein synthesis. Up-regulated ERO1α allows for efficient processing of protein oxidative folding while avoiding the accumulation of immature proteins. On the other hand, the potential ability of ERO1α to utilize alternative electron acceptors instead of oxygen contributes to the protein synthesis of tumor cells under hypoxia. Additionally, it has been reported that ERO1α not only directly promotes the expression of PD-L1 on tumor cells by increasing its oxidative folding, but also indirectly through HIF-1α, thereby inducing T-cell dysfunction [59]. Similarly, Liu et al. found that knockout of ERO1α in tumor cells promoted the infiltration of CD8^+^ T cells and enhanced responses to anti-PD-1 treatment [97]. Furthermore, studies also showed that ERO1α levels were negatively correlated with the sensitivity to immune checkpoint inhibitors (ICIs) [64, 97, 111]. So far, the relationship between ERO1α and other immune checkpoint pathways, such as T-cell immunoglobulin and mucin domain 3 (TIM-3), lymphocyte activation gene 3 (LAG-3) and cytotoxic T-lymphocyte associated protein 4 (CTLA-4), have not been reported.

For myeloid-derived cells, ERO1α has been reported to improve the chemotaxis of MDSCs. Tsutomu et al. reported that the secretion of chemokines granulocyte colony-stimulating factor (G-CSF) and C-X-C motif chemokine ligand (CXCL) 1/2 from tumor cells were increased as ERO1α enhanced their oxidative folding, resulting in the promotion of recruitment and induction of polymorphonuclear (PMN)-MDSCs [70]. Moreover, ERO1α was found to affect the infiltration and differentiation of monocytes. Silencing ERO1α in tumor cells facilitated monocyte infiltration and their differentiation into dendritic cells (DCs) in pancreatic cancer [112]. MDSCs in TME are known for their potent immune-suppressive activity, whereas DCs activate T cells by taking up and presenting tumor antigens [113]. In addition, analyses indicated that ERO1α had effects on macrophage polarization [64, 111]. Macrophages are typically classified into two representative types according to their function and activation: classically activated (M1) and alternatively activated (M2) macrophages [114]. TAMs, the macrophages in TME, are considered to possess an M2-like phenotype and favor tumor progression [115, 116]. Database analysis showed that ERO1α expression in tumor cells was positively correlated with M2 macrophages while negatively correlated with M1 macrophages [64]. Single-cell RNA-sequencing (scRNA-seq) analysis from ERO1α^KO/WT^ mouse model also demonstrated that ERO1α promoted a phenotype transition of TAMs from M1 to M2 type [111]. However, most of these works are observational studies, and there is a need for a more comprehensive dissection of the role of ERO1α in the infiltration, differentiation, and functional execution of immune cells, as well as the underlying mechanisms.

For T cells, it has been confirmed that ERO1α in tumor cells instigated the dysfunction of CD8^+^ T cells, which was characterized by increased exhausted markers (Lag3, Havcr2 and Odcd1) and decreased ability of proliferation, degranulation and secretion of inflammatory cytokines [111]. Mechanistically, ERO1α in tumor cells was revealed to promote the transmission of ER stress to T cells, triggering the CHOP-dependent apoptosis and resulting in dysfunction of T cells [97, 111]. In addition, deletion of ER stress in T cells restrained in vivo tumor growth and restored the sensitivity to ICIs [97]. However, besides being transmitted from tumor cells, ER stress can also be induced in T cells when tumor antigens are submitted to T cells, resulting in a huge protein synthesis burden. Katie et al. reported that the ER stress in CD8^+^ T cells up-regulated ERO1α expression by the PERK/ATF4/CHOP branch of the UPR, in which ERO1α was identified as a key downstream effector of ATF4/CHOP to promote global protein synthesis [117]. Nevertheless, the high level of H_2_O_2_ resulting from up-regulated ERO1α overloaded the processing capability of cells, ultimately leading to mitochondrial exhaustion of CD8^+^ T cells [117]. Of interest, tumor cells and T cells show distinct fates when they encounter up-regulated ERO1α and H_2_O_2_. Though, tumor cells possess a more potent antioxidant capacity than T cells [53, 118]. Whether their ability to reduce oxidizing agents, such as H_2_O_2_, is sufficient to control cell fate (i.e., die or survive) remains in question [53, 119].

Taken together, the up-regulation of ERO1α is a crucial adaptive mechanism by which tumor cells respond to unfavorable microenvironment. ERO1α contributes to the formation of a tumor-supporting immunosuppressive microenvironment by affecting the recruitment and differentiation of immune cells, triggering the dysfunction of T cells, and regulating the PD-1/PD-L1 pathway.

#### Glucose metabolism

It is widely known that aerobic glycolysis is the main pathway of energy metabolism in tumor cells (i.e., the Warburg effect), as well as the pentose phosphate pathway (PPP) [120, 121]. ERO1α has been demonstrated to promote aerobic glycolysis in pancreatic cancer and cervical cancer [68, 89]. In the study of pancreatic cancer, ERO1α was found to promote tumor growth *via* enhancing aerobic glycolysis, whereas inhibition of aerobic glycolysis partially abrogated the supportive effects of ERO1α on tumor growth [68]. Mechanistically, H_2_O_2_ was identified as the mediator for the effects of ERO1α on aerobic glycolysis. However, it remains unclear whether ERO1α can directly regulate the aerobic glycolysis process, and warrants further investigation. Aerobic glycolysis supplies abundant metabolic intermediates, such as glucose-6-phosphate (G-6-P) for the PPP to produce reduced nicotinamide adenine dinucleotide phosphate (NADPH) and GSH, two essential reductants for H_2_O_2_. Therefore, ERO1α confers upon tumor cells an augmented antioxidant capacity relative to normal cells by promoting aerobic glycolysis. In addition to its impacts on aerobic glycolysis and PPP, further studies to explore the regulatory roles of ERO1α in other antioxidant systems are needed.

#### Chemoresistance

Hypoxia and ER stress have been well-documented as major contributors to the chemotherapy resistance of tumor cells, by multiple mechanisms including apoptosis inhibition, metabolic rewiring, anti-oxidant defences and drugs efflux [79]. Meanwhile, hypoxia-/ER stress-induced ERO1α also shows contribution to the chemoresistance of tumor cells. In gastric cancer, silencing ERO1α rendered tumor cells more sensitive to 5-Flourouracil (5-FU) and paclitaxel, suggesting a chemoresistance role of ERO1α in tumor cells [100]. In a breast cancer study, ERO1α inhibition was reported to blunt the tumor resistance to paclitaxel by down-regulating UPR [122]. Numerous anti-tumor drugs, including 5-FU and paclitaxel, have been shown to act by inducing lethal ER stress in tumor cells [20, 123, 124]. Mechanically, increased susceptibility of tumor cells to ER stress upon ERO1α inhibition may explain the drug-resistant role of ERO1α [111]. Furthermore, ERO1α was also shown to undermine anti-tumor immunity by inducing PD-L1 on tumor cells [64, 97, 111].

#### Cell survival

ERO1α was demonstrated to rescue tumor cells from death under ER stress or therapeutic interventions. Ablation of ERO1α resulted in hyper-activation of PERK and an imbalance between IRE1a and PERK, leading to tumor cells apoptosis *via* the CHOP and Caspase-12 pathways [111]. Similarly, in a colon cancer research, deletion of ERO1α was found to promote tumor apoptosis *via* the miR-101/EZH2/Wnt/β-catenin pathway [125]. In addition to apoptosis, ERO1α was also associated to immunogenic cell death (ICD). In lung cancer, ERO1α deletion triggered lethal ER stress in tumor cells and promoted host anti-tumor immunity *via* ICD [111]. However, it is unclear whether ERO1α is related to other tumor cell death modes, such as autophagy, ferroptosis, pyroptosis and necroptosis.

#### Prognostic significance

**Fig. 3:**
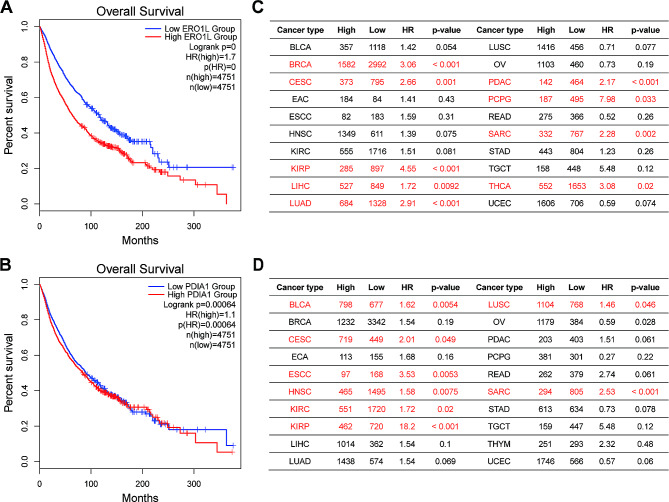
The pan-cancer prognostic value of ERO1α and PDI. Integrated Kaplan-Meier curves from the GEPIA2 database showing the prognostic effect of ERO1α **(A)** and PDI **(B)** expression with patients’ survival across 33 types of cancers. Prognostic analyses from the Kaplan-Meier Plotter database indicating the correlations of ERO1α **(C)** and PDI **(D)** with survival in specific cancers. ERO1α, endoplasmic reticulum oxidoreductase 1 alpha; PDIA1: protein disulfide isomerase A1; HR: hazard ratio. BLCA, Bladder urothelial carcinoma; BRCA, Breast invasive carcinoma; CESC, Cervical squamous cell carcinoma and endocervical adenocarcinoma; ECA, Esophageal adenocarcinoma (EAC); ESCC, Esophageal squamous cell carcinoma; HNSC, Head and neck squamous cell carcinoma; KIRC, Kidney renal clear cell carcinoma; KIRP, Kidney renal papillary cell carcinoma; LIHC, Liver hepatocellular carcinoma; LUAD, Lung adenocarcinoma; LUSC, Lung squamous cell carcinoma; OV, Ovarian serous cystadenocarcinoma; PDAC, Pancreatic ductal adenocarcinoma; PCPG, Pheochromocytoma and paraganglioma; READ, Rectum adenocarcinoma; SARC, Sarcoma; STAD, Stomach adenocarcinoma; TGCT, Testicular germ cell tumors; THCA, Thyroid carcinoma; THYM, Thymoma; UCEC, Uterine corpus endometrial carcinoma

The high expression of ERO1α implies the clinical significance of ERO1α in tumor patients. Data obtained from online databases and clinical follow-up showed that high level of ERO1α in patients was negatively correlated with overall survival (OS), as well as recurrence-free survival (RFS) and disease-free survival (DFS). In addition, results from multivariate Cox regression analysis revealed that high level of ERO1α was also recognized as an independent prognostic factor in breast cancer, lung cancer, pancreatic cancer and bile duct cancer (Table 2). We retrieved prognostic data of ERO1α in tumor patients from public online databases. From the Gene Expression Profiling Interactive Analysis 2 (GEPIA2) database, an integrated result showed that ERO1α expression was negatively associated with patients’ overall survival (OS) with a hazard ratio (HR) of 1.7 (*p* < 0.0001) across 33 cancer types (Fig. 3A). Specifically, data from the Kaplan-Meier Plotter database showed that ERO1α was identified as an indicator of poor prognosis in 9 out of 20 different cancer types (Fig. 3C). Furthermore, we also analyzed the prognostic value of PDIA1, the canonical member of the PDI family members and the major substrate of ERO1α, in tumor patients and showed similar results to ERO1α (Fig. 3B and D).

In addition, ERO1α was also included into multi-gene models as a predictor for poor prognosis of tumor patients. Differentially expressed genes (DEGs) between tumor patients and normal individuals were computationally identified and then screened to construct risk score models. In these models, ERO1α was found to be associated with poor prognosis and was proposed for the prognosis prediction in lung cancer [126–128] and pancreatic cancer [129].

#### Other tumoral-favoring effects of ERO1α

In lung cancer, ERO1α promoted IL-6 receptor (IL-6R) secretion by promoting oxidative folding, and increased soluble IL-6R in turn led to the activation of NF-κB [65]. IL-6 and NF-κB are two well-known effectors to be involved in tumor initiation and progression [130, 131]. Since the availability of public databases has allowed researchers to explore different perspectives of cancer biology, one can recognize that the ERO1α protein is also present in tumor-derived exosomes of bladder, liver and squamous cell carcinomas (retrieved from the ExoCarta database). Thus, providing a new avenue to understand the exosome biology behind ERO1α in tumors.

**Fig. 4:**
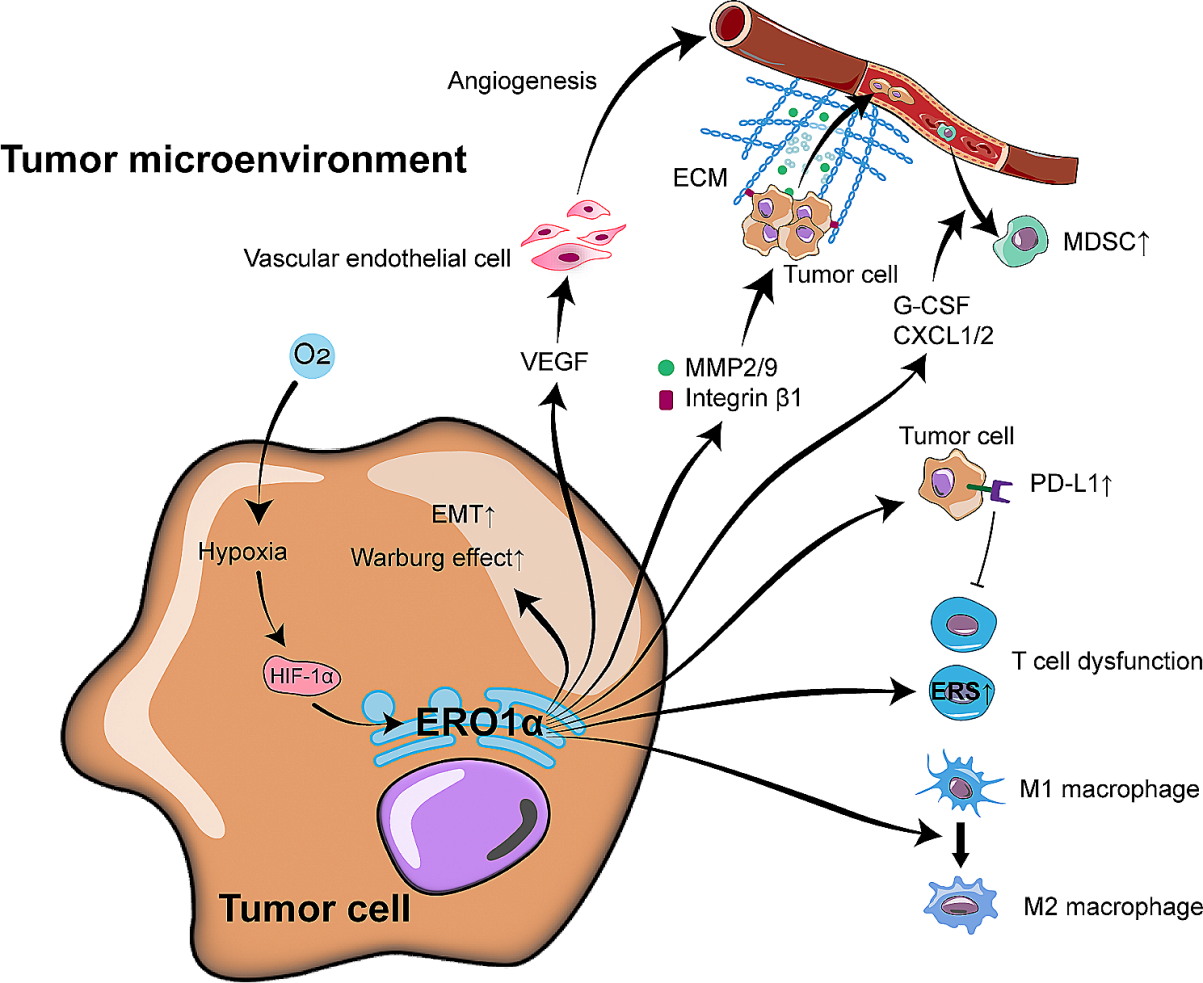
The regulation and the oncogenic roles of ERO1α on tumors. For the regulation of ERO1α, only hypoxia is shown in this figure. In the TME, hypoxia enhances ERO1α via up-regulating HIF-1α. ERO1α not only endows tumor cells with an aggressive phenotype and promotes aerobic glycolysis of tumor cells, but also contributes to induce an immunosuppressive TME by activating immunosuppressive cells while inhibiting immunocompetent cells. ERO1α, endoplasmic reticulum oxidoreductase 1 alpha; PDI, protein disulfide isomerase; EMT, epithelial-mesenchymal transition; VEGF, vascular endothelial growth factor; MMP, matrix degrading enzyme; ECM, extracellular matrix; MDSC, myeloid-derived suppressor cell; G-CSF, granulocyte colony-stimulating factor; CXCL1/2, C-X-C motif chemokine ligand 1/2. PD-L1, programmed cell death ligand-1

Taken together, ERO1α shows a lot of versatility on both tumor cells and TME (Fig. 4). It not only endows tumor cells with faster growth and aggressive phenotype, but also induces an immunosuppressive TME by improving the angiogenesis, the recruitment and differentiation of immunosuppressive cells, and by causing dysfunction of favorable immune cells. Meanwhile, further investigations to unveil a comprehensive landscape of the effects of ERO1α on immune cells in TME are warranted. The impact of ERO1α on tumor cells primarily hinges on enhanced oxidative protein folding, which can be compensated by other redundant oxidoreductases, as mentioned above [42–44]. However, current in vivo and in vitro experiments did show a significant reduction in the expression of ERO1α target genes, such as VEGF, PD-L1, HIF-1α and MMPs, upon the inhibition of ERO1α, suggesting these compensatory counterparts may be impaired. Therefore, to reveal how tumor cells coordinate ERO1α and its compensatory mechanisms becomes an intriguing avenue of exploration.

### Targeting ERO1α for anti-tumor treatment

**Fig. 5:**
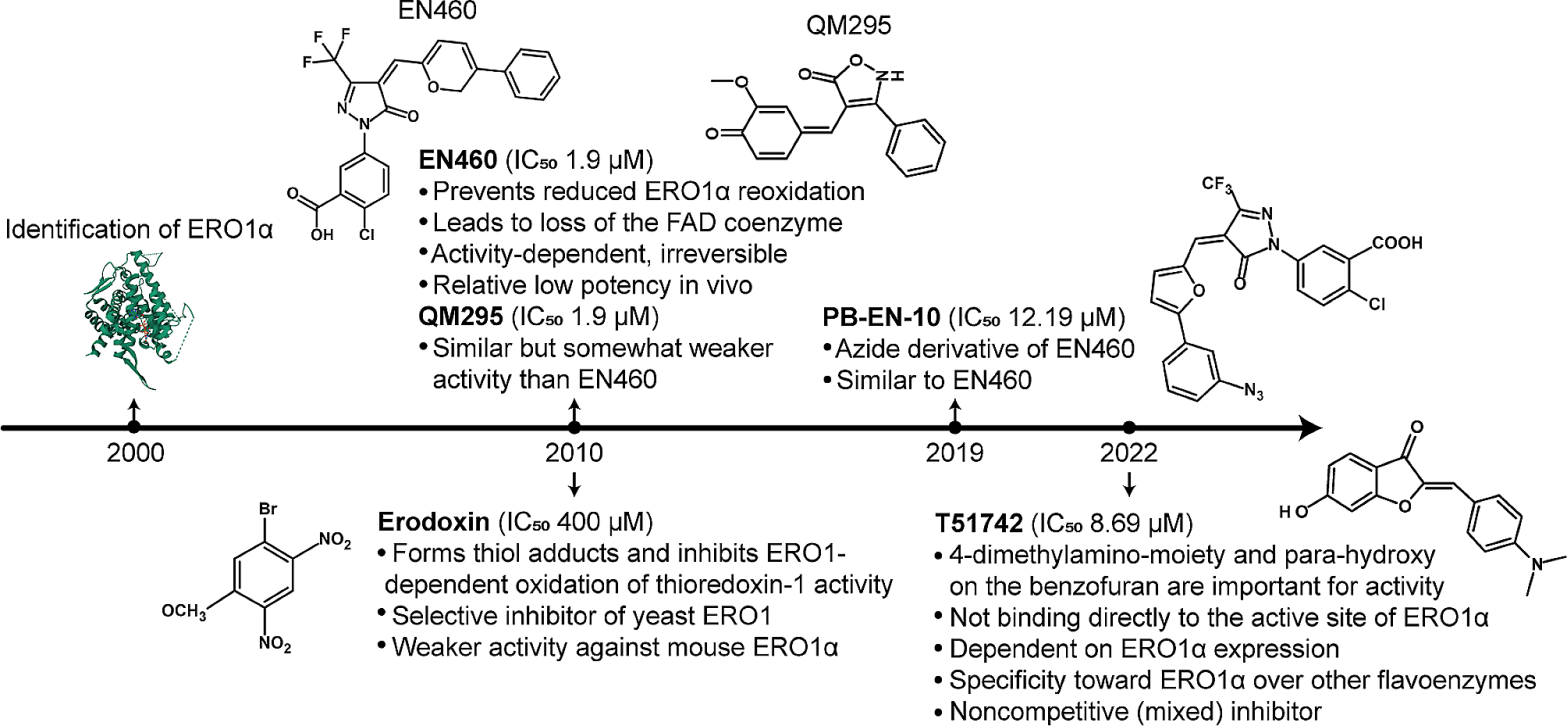
Timeline of the ERO1α inhibitors

Despite accumulating evidence that ERO1α exerts a profound influence on tumors and would be an attractive target for anti-tumor therapy, few pharmacological inhibitors are available for further validation and none are approved for clinical use. The challenge predominately arises from the highly conserved structure of the FAD cofactor-binding domain across enzymes, suggesting that inhibitors not only recognize the FAD domain in ERO1α, but also other FAD-containing enzymes, such as lysine specific demethylase 1 (LSD1), monoamine oxidases A and B (MAO-A and MAO-B) [132]. To date, several compounds have been reported to target ERO1α in mammals (Fig. 5). EN460 and QM295 stand as the first two identified ERO1α inhibitors through a biochemical high-throughput screen and have been shown to interact with reduced ERO1α and prevent re-oxidation [133]. PB-EN-10 is an azide derivative of EN460 and shows similar effects [132]. Erodoxin, a dinitrobromobenzene compound, acts as a selective inhibitor of yeast ERO1, but has somewhat weaker activity against mouse ERO1α [133, 134]. However, these inhibitors lack selectivity for ERO1α, and indeed, they inhibit other FAD-containing enzymes as well [132]. Recently, Brennan et al. reported a novel ERO1α inhibitor named T151742, a sulfuretin derivative, showing heightened activity (IC_50_: 8.27µM) compared to EN460 (IC_50_: 16.46µM) and isozyme specificity for ERO1α as compared to that for ERO1β and no detectable binding to the FAD-containing enzyme LSD-1 [135]. However, further investigations are warranted to determine its in vivo efficacy and safety.

Given that PDI directly interacts with ERO1α, targeting PDI would also be a viable approach to block oxidative protein folding. In fact, PDI has also been shown to be up-regulated in a variety of cancer types and exhibit pro-tumoral roles [136]. Various chemical inhibitors of PDI have been identified and some of them showed potential anti-tumor effect [137]. However, the presence of over 20 structurally similar PDI homologues in eukaryotes limits the development of specific inhibitors. Considering the inextricable interplay between ERO1α and PDI, a more effective and specific strategy involves developing inhibitors that disrupt the interaction between ERO1α and PDI. Recently, Zhang et al. reported that valine (Val) 101, a hydrophobic residue in the active site-containing loop of ERO1α, is crucial for the recognition of PDI catalytic domain [63]. Mutation of Val101 weaken the activity of ERO1α in oxidative protein folding, and more importantly, impaired tumor progression. This finding not only provides a reliable target site for inhibitor development, but also a paradigm for targeting the ERO1α-PDI interface.

The highly conserved structure of the FAD-binding domain limits the development of ERO1α inhibitors. In recent years, proteolysis-targeting chimera (PROTAC) has been emerged as a novel technology for targeted protein degradation [138, 139]. PROTAC is a bifunctional molecule consists of three domains: a protein of interest (POI) ligand, a E3 ubiquitin ligase ligand, and a linker which covalently interconnects with these two ligands. Upon binding to the target protein, the PROTAC molecule can recruit E3 ubiquitin ligase for protein ubiquitination, which is subjected to proteasome-mediated degradation [140]. Therefore, with respect to ERO1α, the development of PROTAC molecules does not require targeting the active center of ERO1α, but only the ability to specifically recognize ERO1α protein, which would greatly help to avoid off-target effects of the current ERO1α inhibitors. Notably, however, there are also some challenges for PROTAC to be a successful drug development approach [141].

Strategies to disturb UPR or to increase the protein accumulation in the ER are the current approaches in anti-tumor treatment targeting ER stress [26, 27]. For instance, inhibition of the UPR proteins, such as PERK, IRE1 or eIF2α, has been reported to show anti-tumor properties [26]. Tunicamycin, an antibiotic, has been shown to inhibit the N-glycosylation of proteins in the ER, thereby inducing overwhelming ER stress [142]. In addition, proteasome inhibitors, widely used as anti-tumor drugs (especially in hematological tumors), such as bortezomib, have been shown to induce tumor death by inhibiting proteolysis, thereby increasing protein accumulation in the ER and resulting in lethal ER stress [143]. Given that ERO1α is a crucial player in the ER protein homeostasis, synergistic inhibition of ERO1α and other ER stress-inducing targets mentioned above would be a promising approach in anti-tumor treatment. For example, the combined treatment with proteasome inhibitors, which retards the oxidative folding and proteolysis of proteins concurrently, could induce ER stress more efficiently than their single use. Actually, the synergistic effect of this dual inhibition has been in vitro confirmed. ISRIB, a small molecule that inhibits the phosphorylation of eIF2α and removes its inhibition on global protein translation, was found to synergistically interact with the genetic deficiency of ERO1α and to impair breast tumor growth and spread [122]. However, the in vivo availability and utility of the dual inhibition strategy remain unclear, given the current absence of clinically available ERO1α inhibitors. Therefore, the development of highly specific and efficient ERO1α-targeting drugs is a critical objective.

### A few key considerations about ERO1α

As mentioned earlier, reports on ERO1α expression as a prognostic indicator in various cancers raise a few important questions: (1) can we target ERO1α without affecting other FAD-containing enzymes, (2) how ERO1α affects the response of cancer immunotherapies, and is there a synergistic effect of the combination treatment with other known ER stress/UPR targeting drugs, (3) ERO1α-PDI interactions have been known for years, and while both are of central importance, it is still difficult to determine which one predominates. Given that both ERO1α and PDI are overexpressed in tumors and their close interplay, can we rule out the possibility that targeting ERO1α might also act by affecting PDI, and how this differs from directly targeting PDI, (4) which cancer immunotherapy approach would benefit from the combined treatment with ERO1α inhibition. Cytokine-induced killer (CIK) cell immunotherapy has been successfully demonstrated to reinforce immune system to fight against tumors due to its attributes such as non-toxic, heterogeneous cell population (T cells, NKT cells and NK cells) and synergistic compatibility with ICIs [144]. Therefore, in our opinion, CIK cell immunotherapy may represent an opportunity in this setting.

Though we mainly focus on cancer, it is worth mentioning that ER-related dysregulation (especially involving ERO1α) has also been found in other diseases such as diabetes [145, 146], neurodegenerative diseases (e.g., Parkinson’s disease [147], Alzheimer’s disease [148, 149], Huntington’s disease [150] and amyotrophic lateral sclerosis [151, 152]), and cardiovascular diseases [153–155]. Therefore, it is important to gain more comprehensive insights into the involvement of disease-specific genetic/epigenetic processes and cellular mechanisms affecting ERO1α in general.

**Table 1:** Expression profile of ERO1α in tumors

Cancer Type	Ref.	Data source	Positive group	Normal Control	ERO1A expression
Bile duct cancer	[62]	Cell line	Tumor cell lines (*n*=5)	Bile duct epithelial cell line (*n*=1)	↑ (Protein-WB)
		Tissue	Tissue microarray (*n*=186)	Adjacent normal tissues (*n*=36)	↑ (Protein-IHC)
Cervical cancer	[63]	Tissue	Patient tumor tissues (*n*=15)	Adjacent normal tissues (*n*=15)	↑ (Protein-WB)
		Tissue	Tissue microarray (*n*=69)	Normal cervical tissues (*n*=9)	↑ (Protein-IHC)
Lung cancer	[64]	Database	Patient tumor tissues (*n*=376, Oncomine)	Normal tissues (*n*=150)	↑ (mRNA)
		Database	Patient tumor tissues (*n*=483, TCGA)	Normal tissues (*n*=59)	↑ (mRNA)
		Database	Patient tumor tissues (*n*=483, TCGA + GTEx)	Normal tissues (*n*=347)	↑ (mRNA)
		Database	Patient tumor tissues (*n*=356, GEO)	Normal biopsies (*n*=170)	↑ (mRNA)
	[65]	Tissue	Tissue microarray (*n*=80)	Adjacent normal tissues (*n*=80)	↑ (Protein-IHC)
	[66]	Tissue	Patient tumor tissues (*n*=102)	Adjacent normal tissues (*n*=102)	↑ (Protein-IHC)
		Cell line	Tumor cell lines (*n*=4)	Lung epithelial cell line (*n*=1)	↑ (Protein-WB)
	[67]	Database	Patient tumor tissues (*n*=502, TCGA)	Normal tissues (*n*=49)	↑ (mRNA)
Pancreatic cancer	[68]	Database	Patient tumor tissues (*n*=179, TCGA + GTEx)	Normal tissues (*n*=171)	↑ (mRNA)
		Database	Patient tumor tissues (*n*=96, GEO)	Normal tissues (*n*=122)	↑ (mRNA)
		Tissue	Tissue microarray (*n*=205)	Adjacent normal tissues (*n*=205)	↑ (Protein-IHC)
	[69]	Database	Patient tumor tissues (*n*=145, GEO)	Normal tissues (*n*=46)	↑(mRNA)
		Tissue	Patient tumor tissues (*n*=8)	Normal tissues (*n*=3)	↑ (mRNA & protein, qPCR & WB)
		Cell line	Tumor cell lines (*n*=6)	Pancreatic epithelial cell line (*n*=1)	↑ (mRNA & protein, qPCR & WB)
Breast cancer	[70]	Cell line	Tumor cell lines (*n*=7)	Normal tissue (*n*=1)	↑ (mRNA, qPCR)
Prostate cancer	[71]	Tissue	Patient tumor tissues (*n*=12)	Normal tissues (*n*=6)	*NS* (protein, WB)
		Cell line	Tumor cell lines (*n*=4)	Epithelial prostate cell line (*n*=1)	*NS* (protein, WB)
Liver cancer	[58]	Tissue	Patient tumor tissues (*n*=114)	Adjacent normal tissues (*n*=114)	↑ (mRNA & protein, qPCR & WB & IHC)
		Database	Patient tumor tissues (*n*=371, TCGA)	Normal tissues (50)	↑ (mRNA)
		Cell line	Tumor cell lines (*n*=5)	Liver cell line (*n*=1)	↑ (mRNA & protein, qPCR & WB & IHC)
Gastric cancer	[72]	Tissue	Patient tumor tissues (*n*=105)	Adjacent normal tissues (*n*=105)	↑ (mRNA & protein, qPCR & WB & IHC)

Duplicate data in different references was deleted and only one was retained. *NS*, no significance; GEO, Gene Expression Omnibus; TCGA, The Cancer Genome Atlas Program; GTEx, Genotype-Tissue Expression; WB, Western blot;IHC, Immunohistochemistry; qPCR, quantitative polymerase chain reaction.

**Table 2:** The implications of ERO1α in tumors

**Cancer type**	**Ref.**	**Biological behavior**	**EMT**	**Angiogenesis**	**Xenograft in mice**	**Prognostic significance (Patients with ERO1A +/high)**
Breast cancer	[93]	-	-	-	-	RFS↓
	[87]	-	-	CD31(KD↓, OE↑), HUVEC(OE↑)	Lung metastasis (KD↓), OS (KD↓)	-
	[70]	KD(*NS*)	-	-	Tumor growth (KD↓, OE↑), OS (KD↑, OE↓)	-
	[94]	-	-	CD31 (KD↓, OE↑)	Tumor growth (KD↓, OE↑)	OS↓, #
	[95]	-	-		Tumor growth (KD↓), lung metastasis (KD↓)	DFS↓, OS↓, #
	[96]	KO↓	-	HUVEC (KO↓)	Tumor growth (KO *NS*), lung metastasis (KO↓)	OS (*NS*)
Lung cancer	[97]	-	-	-	-	RFS↓
	[64]	-	-	-	-	RFS↓, OS↓, DFS↓
	[65]	KD↓, OE↑	KD↓, OE↑	-	Tumor metastasis (KD↓, OE↑)	RFS↓, OS↓
	[67]	KD↓	-	-	-	OS↓
	[66]	KD↓, OE↑	-	-	Tumor growth (KO↓)	-
	[68]	-	-	-	-	DFS↓, #
Bile duct cancer	[63]	KD↓, OE↑	KD↓, OE↑	-	-	DFS (*NS*), OS↓, #
Prostate cancer	[71]	KD↓	-	-	-	-
Pancreatic cancer	[99]	KD↓	-	-	Tumor growth (EN460↓),	OS↓, DFS ↓
					liver metastasis (EN460↓), OS (EN460↓)	
	[69]	KD↓, OE↑	-	-	Tumor growth (KD↓, OE↑)	-
	[68]	KD↓, OE↑	-	-	Tumor growth (KD↓, OE↑)	OS↓, #
Gastric cancer	[100]	KD↓	-	-	-	RFS↓, OS↓
Colon cancer	[101]	KD (*NS*)	KO↓	-	Tumor growth (KO↓)	-
Liver cancer	[58]	KD↓, OE↑	KD↓, OE↑	HUVEC (KD↓, OE↑), CD34 (KD↓, OE↑)	Lung metastasis (KD↓, OE↑)	RFS↓, OS↓
Cervical cancer	[63]	KO↓	KO↓	-	Tumor growth (KO↓)	OS↓

Duplicate data in different references was deleted and only one was retained. EMT, epithelial–mesenchymal transition; *NS*, no significance; KD, knockdown; KO, knockout; OE, overexpression; EN460, ERO1α inhibitor; HUVEC, human umbilical vein endothelial cells; OS, overall survival; RFS, recurrence-free survival; DFS, disease-free survival; #, Independent prognostic factor; -, none.

### Concluding remarks

ERO1α plays a role for tumor support, and targeting ERO1α holds promise as an antitumor strategy. Besides, the dual characteristics of ERO1α, i.e., flexibility to ER stress in tumors and modulation with immunosuppressive TME, make it a strong candidate for future research on its crucial adaptive mechanisms. Certainly, with the advent of new technologies, the peculiar way of molecular recognition of ERO1α in the cancer landscape is awaited.

## Data Availability

Not applicable.

## Abbreviations

ATF4/6: Activating transcription factor4/6
CAFs: Cancer-associated fibroblasts
CaMKII: Calcium/calmodulin-dependent protein kinase II
CA9: Carbonic anhydrase 9
CCL: C-C chemokine ligand
CHIP: Chromatin immunoprecipitation
CHOP: C/EBP homologous protein
CIK cell: Cytokine-induced killer cell
CSCs: Cancer stem cells
CTLA-4: Cytotoxic T-lymphocyte associated protein 4
CXCL: C-X-C motif chemokine ligand
DCs: Dendritic cells
DEGs: Differentially expressed genes
DFS: Disease-free survival
eIF2α: Eukaryotic initiation factor 2α
DNMT1: DNA methyltransferase 1
ECM: Extracellular matrix (ECM)
E_GSH_: Glutathione reduction potential
EHMT2: euchromatic histone-lysine N-methyltransferase 2
EMT: Epithelial-mesenchymal transition
ERO1α: Endoplasmic reticulum oxidoreductase 1 alpha
ER: Endoplasmic reticulum
ERAD: ER-associated degradation
FAD: Flavin adenosine dinucleotide
G-6-P: Glucose-6-phosphate
G-CSF: Granulocyte colony-stimulating factor
GEO: Gene Expression Omnibus
GEPIA2: Gene Expression Profiling Interactive Analysis 2
GPx8: Glutathione peroxidase 8
GSH: Glutathione
GTEx: Genotype-Tissue Expression
HIF-1α: Hypoxia-inducible factor-1 alpha
HR: Hazard ratio
HUVEC: Human umbilical vein endothelial cells
ICD: Immunogenic cell death
ICIs: Immune checkpoint inhibitors
IP3R: Inositol 1,4,5-triphosphate receptor
IRE1: Inositol requiring enzyme 1
JNK: c-Jun N-terminal kinase
LAG-3: Lymphocyte activation gene 3
LSD-1: lysine specific demethylase 1
MAO-A/B: Monoamine oxidases A/B
MDSCs: Myeloid-derived suppressor cells
MMPs: Matrix degrading enzymes
NADPH: Nicotinamide adenine dinucleotide phosphate
NFIB: Nuclear factor IB
NK cells: Natural killer cells
NOX: NADPH oxidase
OS: Overall survival
PDHs: Prolyl hydroxylases
PD-L1: Programmed cell death ligand-1
PDI: Protein disulfide isomerase
PERK: Protein kinase R (PKR)-like endoplasmic reticulum kinase
PMN-MDSCs: Polymorphonuclear-MDSCs
POI: Protein of interest
PPP: Pentose phosphate pathway
PRDX4: Peroxiredoxin 4
PROTAC: Proteolysis-targeting chimera
PrxIV: peroxiredoxin IV
RFS: Recurrence-free survival
RyR: Ryanodine receptor
TAM: Tumor associated macrophage
TCGA: The cancer genome atlas program
TIM-3: T-cell immunoglobulin and mucin domain 3
TME: Tumor microenvironment
UPR: Unfolded protein response
VEGF: Vascular endothelial growth factor
VKOR: vitamin K epoxide reductase
XBP1: X-box protein 1
5-FU: 5-Flourouracil
